# Paid Organ Donation: Case Report and Review of the Literature on Health Implications for Kidney Donors and Recipients

**DOI:** 10.3390/pathogens14080819

**Published:** 2025-08-19

**Authors:** Małgorzata Marchelek-Myśliwiec, Krzysztof Korzeniewski, Emilia Marchelek, Joanna Stępniewska, Danuta Kosik-Bogacka

**Affiliations:** 1 Clinic of Nephrology, Transplantology and Internal Medicine, Pomeranian Medical University in Szczecin, Powstańców Wielkopolskich 72, 70-111 Szczecin, Poland; malgorzata.marchelek.mysliwiec@pum.edu.pl (M.M.-M.); joanna.stepniewska@pum.edu.pl (J.S.); 2 Department of Epidemiology and Tropical Medicine, Military Institute of Medicine—National Research Institute, Szaserów 128, 04-141 Warsaw, Poland; kkorzeniewski@wim.mil.pl; 3 School of Medicine and Dentistry, Faculty of Clinical and Biomedical Sciences, University of Central Lancashire, Preston PR1 2HE, UK; marchelekemilia@gmail.com; 4 Department of Biology, Parasitology and Pharmaceutical Botany, Pomeranian Medical University in Szczecin, Powstanców Wielkopolskich 72, 70-111 Szczecin, Poland

**Keywords:** tourism transplantation, kidney transplantation, transplant tourism, infection

## Abstract

The shortage of organs for use in transplantation has contributed to the development of an international commercial market for organ transplantation. Unfortunately, transplant tourism (TT) is associated with risks for surgical complications, poor graft outcome, increased mortality, and infectious complications. TT increases the risk of several viral (HIV and hepatitis B and C viruses), bacterial (*Mycobacterium tuberculosis, Pseudomonas* sp., *Enterococcus* sp., *Escherichia coli,* and *Acinetobacter* sp.), fungal (*Aspergillus* sp., Zygomycetes, *Ramichloridium* sp., *Scedosporium apiospermum*, and *Trichosporon* sp.), and parasitic (*Plasmodium* sp., *Trypanosoma cruzi* and *Strongyloides* sp., and *Microsporidia* sp.) infections. This paper presents a case report of an anonymous patient who travelled to Pakistan and underwent a commercial kidney transplant. He developed infection from extended-spectrum β-lactamase-producing *Escherichia coli* (ESBL-EC). Moreover, we reviewed all published cases of bacterial, viral, and fungal infections in kidney transplant recipients who bought their organs abroad.

## 1. Introduction

**Figure 1:**
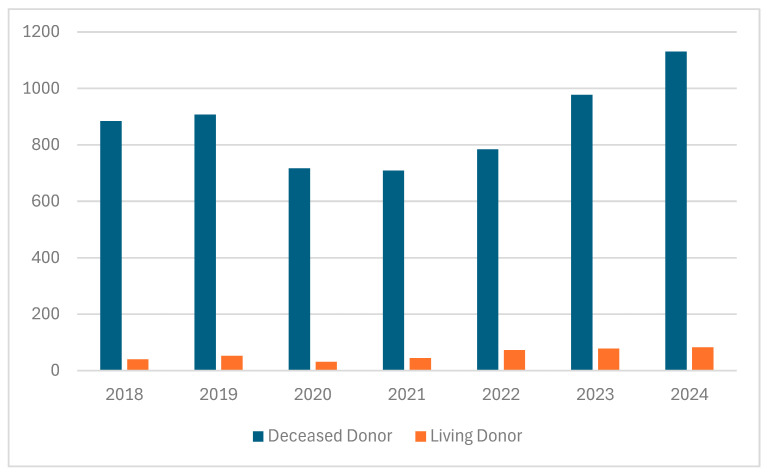
Number of kidney transplants in Poland from deceased and living donors (2018–2024) [9].

The demand for organs is rapidly increasing due to ongoing demographic changes, which are associated with a higher incidence of heart, lung, liver, and kidney diseases caused by various nutritional and behavioral factors, and the development of diagnostic tools, enabling the early diagnostic of cardiac, liver, kidney, or bone marrow pathologies [1]. Although many countries have taken numerous steps to increase the number of transplanted organs from related or deceased donors, the shortage of organs for transplantation remains a significant problem [2]. Spain is the leading European country in terms of transplantation rates, with 40–50 kidney transplants performed per one million inhabitants, a figure that is approximately 4–5 times higher than that observed in Poland [3]. One of the reasons for the shortage of organs available for transplantation is religious and cultural perspectives on organ donation. Beliefs, particularly in Asian regions, play a crucial role in attitudes and decision-making regarding organ donation. Christianity, Hinduism, Sikhism, and Buddhism generally support organ donation [4,5]. In the Islamic Encyclopedia of Fatwas, it was declared that there is no objection to organ donation; however, it must be subject to several conditions. These include first-person authorization, that donation occur either while living or after circulatory declaration of death, respect for the donor himself, a guarantee that his quality of life will remain at the level before donation, and that reproductive organs are not donated, among other conditions [6,7,8]. According to Jewish law, it is prohibited to desecrate or mutilate the dead and prohibited to derive benefit from the dead body; there is a positive commandment to bury the dead body intact. Harvesting organs from the dead violates all these prohibitions. However based on the commandment to choose life, the rabbinic scholars rule that the obligation to pikuach nefesh (save life), overrides almost all religious laws [8]. In Poland, despite the efforts of the medical community, the annual number of kidney transplants remains insufficient (Figure 1).

This has led to the emergence of organ trafficking and transplant tourism (TT) [10]. The main organ importers are developed countries, while the main organ exporters are developing countries [11]. Transplant tourism is associated with many risks, including health and life hazards for both donors and recipients [12]. Several up-to-date reports have described significantly worse short- and long-term outcomes in TT patients, in comparison to those who received organs transplants in their country of residence [2,12,13,14,15,16]. The one-year survival rate of kidneys transplanted through TT was 88%, compared to 97% in patients who received transplants locally. In a longer-term follow-up that lasted 8 years, these rates were 72% in the TT group versus 94% in the locally transplanted group [14]. TT also presents a significant ethical issue, as organ trafficking is associated with coercion, exploitation, human trafficking, and non-standard medical practices. Donors are most often individuals living in extreme poverty, who are forced to sell organs due to financial hardships [13]. For those reasons, TT is considered a practice that violates international laws, including the Convention on Human Rights and Biomedicine [17]. The only country where paid organ donation is legal and regulated by specific legal provisions since 1988 is Iran [18]. Countries such as Pakistan, India, China, and the Philippines have widespread black markets for organ trade, even though local regulations prohibit commercial organ sales [19]. It is estimated that TT covers around 10% of all available organs, mostly kidneys [1,2,11,20]. The exact number of people who have paid for transplanted organs is difficult to determine due to the illegal nature of organ trafficking. Therefore, in 2008, the Istanbul Declaration was signed at the International Summit on Transplant Tourism. It proclaimed the fight against and prohibition of organ trafficking and protection of the poorest individuals from being exploited in organ trade, as well as establishing guidelines based on the highest ethical standards for organ donation and transplantation [21]. In Poland, the Act on the Procurement, Storage, and Transplantation of Cells, Tissues, and Organs of 1 July 2005 (Journal of Laws 2005 No. 169, item 1411) regulates matters related to transplantation. This act, among others, outlines the rules for procuring organs from living donors. A transplant from a living donor who is not a relative must be authorized by the transplant commission and a consent from the court must be obtained. A person who purchases an organ in countries where this practice is common and legal is not considered a criminal and will not be criminally prosecuted in Poland. The median wait times for deceased donor kidney transplantation in Poland is around 427 days [9]. Thus, some patients perceive the purchase of an organ abroad as a realistic alternative to prolonged waiting for a transplant; however, there is a lack of data regarding their awareness of the potential health risks involved. Due to economic conditions in Central Europe, including Poland, TT is not widely evident yet. This stands in contrast to Western European countries, where higher income levels have enabled patients to seek treatment abroad, which ultimately leads to increased rates of reported TT.

The aim of this publication is to draw the attention of the medical community in Central European countries to the potential occurrence of transplant tourism and to emphasize the need to prepare for possible complications arising from transplantations performed outside the national healthcare system. The structure of this present study is twofold. First, it presents the first such case reported in Poland, the anonymous case of a man who traveled overseas for a kidney transplant resulting in an extended-spectrum β-lactamase-producing *Escherichia coli* (ESBL-EC) infection. Second, we reviewed all published cases of bacterial, viral, and fungal infections in kidney transplant recipients who bought their organs abroad.

## 2. Case Report

A patient presented to the Clinic of Nephrology, Transplantology and Internal Medicine at the Pomeranian Medical University in Szczecin (PUM) due to persistent symptoms of severe weakness lasting for two weeks, which significantly impaired his mobility. The patient reported having undergone a kidney transplant procedure two weeks earlier in Pakistan. Prior to surgery, he had been maintained on chronic hemodialysis for 9 months. His condition during dialysis remained stable and did not necessitate an expedited transplantation for medical reasons. In Poland, he had been on the national kidney transplant waiting list for the preceding 10 months. The patient was aware of the potential risks associated with undergoing transplantation abroad but prioritized rapid access to the procedure over the possibility of complications. This case involved a patient of high socio-economic status, for whom the cost of transplantation was not a significant limiting factor. The choice of destination was determined by the short waiting time for surgery. In discussion with a psychologist, he admitted that he had approached the entire situation in a purely business-like manner.

The documentation on the patient’s surgery was minimal, containing only his personal details, the date of the transplant, and the prescribed immunosuppressive medications: cyclosporine (CsA) at a dose of 2 × 400 mg, mycophenolate sodium (MPS) at 2 × 360 mg, and the antibiotic ciprofloxacin at 1 × 200 mg. The discharge summary did not include any laboratory test results—neither basic, virological, nor immunological.

The patient reported that prior to the procedure, he was not informed about the exact location or conditions under which the surgery would be performed. Furthermore, during his transport to the surgical facility, he was not able to observe the travel route.

Upon admission to the Clinic of Nephrology, Transplantology and Internal Medicine at PUM, clinical examination revealed signs of dehydration and a systolic murmur in the mitral valve auscultation area. Laboratory tests showed elevated creatinine levels—2.98 mg/dL, with an estimated GFR of 21 mL/min/1.73 m^2^, and an elevated CsA of 663 ng/mL at C0 point (Table 1).

Virological tests for viral hepatitis, human immunodeficiency virus (HIV), cytomegalovirus (CMV), and polyomavirus (BKV) were all negative. Blood and urine cultures were also negative. However, a rectal swab culture revealed colonization with extended-spectrum β-lactamase-producing *Escherichia coli* (ESBL-EC). The urine output was preserved. A kidney biopsy was performed, which revealed no evidence of acute rejection of the transplanted kidney. During the following days of hospitalization, the immunosuppressive regimen was modified and CsA was replaced with tacrolimus (TAC). After an initial improvement in graft function, a subsequent rise in creatinine levels was observed, reaching 5.83 mg/dL. The rise was accompanied by a decrease in urine output to 500 mL/day. Two hemodialysis sessions were performed. A second kidney biopsy was considered; however, after reducing the TAC dose and administering intensive hydration therapy, urine output increased up to 4000 mL/day, and creatinine levels dropped to 2.19 mg/dL. The patient was discharged and referred for continued care at the Department of Nephrology and Transplantology at the Provincial Hospital in Szczecin, where he had previously undergone hemodialysis. Over a 5-year follow-up period, the function of the patient’s transplanted kidney remained stable, with a GFR of approximately 20 mL/min/1.73 m^2^.

## 3. Methods

This study was based on a review of the scientific literature obtained from validated databases, including PubMed, the National Center for Biotechnology Information (NCBI), ScienceDirect, and Google Scholar. The search was performed on DATA using the following keywords: “transplant” AND “kidney” AND “tourism” AND “commercial” AND “infection.” Inclusion criteria encompassed case reports and peer-reviewed research articles published in English. Studies not published in English and gray literature (e.g., conference proceedings and abstracts) were excluded. The literature search was conducted in accordance with the PRISMA guidelines.

## 4. Results

Based on the scientific literature, we identified 16 cases of bacterial, viral, and fungal infections in patients who traveled abroad for renal transplantation (Table 1). The data indicate that viral infections are the most frequently reported, including cytomegalovirus, hepatitis B, and disseminated herpes zoster. Of significant concern are infections of mixed etiology and with multiple localizations. These include infections transmitted along with the transplanted organ, postoperative wound infections, opportunistic infections, and infections resulting from exposure to environments with poor epidemiological standards. Such complications may lead to graft loss and, in the most severe cases, patient death. Individual case descriptions, etiological agents, and transplant outcomes are summarized in Table 2.

## 5. Discussion

Since the mid-1990s, Pakistan has been a popular destination for TT. By 2007, approximately 2000 commercial kidney transplants were performed annually in Pakistan, around 1500 of which were performed on foreign patients, particularly from the Middle East, India, and Europe [27]. These transplants were typically carried out in private hospitals in Punjab, using organs obtained from impoverished local residents [28]. Since 2007, organ trafficking has been classified as a criminal offense under Pakistan’s Transplantation of Human Organs and Tissues Ordinance (2007), followed by the Transplantation of Human Organs and Tissues Act 2010 (Act VI of 2010). Although the rate of illegal kidney transplants initially dropped significantly, it began to rise again due to poor enforcement and corruption [29]. The patient described in our case underwent his transplant procedure in Islamabad.

In the presented case, as well as in previous analyses, poor communication between the transplant center abroad and the center responsible for continuing care in the patient’s home country was identified. This issue is especially problematic due to inadequate documentation regarding the donor and perioperative care [13,14,24,30,31]. In the patient’s case, there was a lack of information about laboratory test results, including the donor’s CMV serological status, HLA matching, cold ischemia time, intraoperative procedures, as well as descriptions of immunosuppressive regimens and antimicrobial prophylaxis, which have also been highlighted in additional scientific literature [31].

The costs of kidney transplantation in EU countries include eligibility assessments, the surgical procedure, hospitalization, immunosuppressive medications, and post-transplant care. In Poland, these costs amount to approximately $12,000 and cover the surgery and treatment during the first 30 days. However, no fees are charged for insured patients. In contrast, in Pakistan, the cost of kidney transplantation can vary depending on the clinic, region, and the individual needs of the patient. The price of the surgery ranges from approximately $10,000 to $25,000. Additionally, there may be extra costs related to preoperative preparation, hospitalization, immunosuppressive drugs, and postoperative care. Transplant tourism carries significant risks due to the lack of appropriate prevention of opportunistic infections, inadequate screening tests upon arrival, and a high number of patients lost throughout the long-term follow-up [32]. A higher incidence of acute kidney rejection was observed in transplant tourists during the first year of follow-up compared to those transplanted in their home countries (27.9% vs. 9.9%), as well as higher average creatinine levels after 6 months (120 vs. 101 µmol/L) and 1 year (113 vs. 98 µmol/L) [33]. Increased frequency of both cellular and humoral acute rejection episodes has also been documented [34,35]. Furthermore, patients who undergo transplants abroad are at an approximately 20-times-higher risk of hospitalization, particularly during the first 3 months after transplantation (about 40%) [36,37]. Causes for hospitalization include, among others, surgical complications such as anastomotic leaks, fistulas, and urinary tract obstructions [38].

In patients who undergo transplantation abroad, viral, bacterial, fungal, and parasitic infections are frequently observed. The risk of infections in patients who undergo transplantation abroad is higher compared to those transplanted locally, with rates of 45–54% versus 5%, respectively [13,14]. High infection rates stem from intraoperative and hospital-acquired infections, or those originating from the donor. These can also include infections acquired during the peri-transplant period while in endemic areas [31]. The living donors used for transplant tourism typically come from low socio-economic groups and are more likely to have infections [39]. Countries that are hubs for transplant tourism are tropical or subtropical regions with high rates of endemic infections, including malaria, tuberculosis, hepatitis E, and viral infections such as Zika and Chikungunya, as well as rabies. Furthermore, these regions also have a high prevalence of pathogens like HIV, HBV, and HCV, making viral infections more common in transplanted individuals. It has been reported that the frequency of HIV and HBV infections related to transplants abroad is 4–6% and 2–12%, respectively [34,38].

The increased number of infections may also be attributed to insufficient screening of donors for infectious diseases, as well as poor hygienic and operative conditions. Furthermore, inadequate patient education regarding the risk of infections after transplantation and the lack of prophylaxis against opportunistic infections may also contribute to the issue [38]. Bacterial infections are often caused by multidrug-resistant strains, including *Pseudomonas* spp. and vancomycin-resistant *Enterococcus* spp. [24]. Infections caused by Gram-negative rods, such as *Klebsiella pneumoniae*, *Escherichia coli*, and *Pseudomonas aeruginosa*, and Gram-positive cocci, including *Staphylococcus aureus* and *Streptococcus pneumoniae*, have also been reported. Additionally, *Legionella pneumophila* and non-tuberculous mycobacteria infections have been described [40]. Opportunistic invasions, including infections caused by *Legionella pneumophila* and non-tuberculous mycobacteria, are also frequently observed [40]. Patients with bacterial infections have an increased risk of developing systemic symptoms, including sepsis and septic shock. Urinary tract and pulmonary infections are particularly common in these patients [41].

In this presented study, the patient who underwent a transplant in Pakistan and returned to Poland was found to have an infection with extended-spectrum β-lactamase-producing *Escherichia coli*. The most commonly reported infectious diseases in Pakistan (data from 2017–2024) include malaria, dengue, cholera, tuberculosis, measles, and COVID-19. In addition, cases of poliomyelitis, diphtheria, pertussis, Chikungunya, and HIV have also been reported in Pakistan.

Another type of complication following commercial renal transplantation is invasive fungal infections, which often occur at the transplant site and are associated with a high rate of graft loss and mortality. These infections are difficult to recognize, diagnose, and treat. In one case, following a kidney transplant in Pakistan, invasive aspergillosis of the allograft vascular anastomosis was reported [25]. Additionally, invasive infections caused by Zygomycetes, *Ramichloridium mackenziei*, and *Scedosporium apiospermum* have also been documented [25,42]. The transmission of fungal infections at the surgical site suggests poor infection control in the operating room [43].

Parasitic infections may also occur, and can be caused, among other things, by blood-derived products and donor organs. These parasitic infections include invasions by *Plasmodium* spp., *Babesia* spp., *Trypanosoma cruzi*, *Schistosoma* spp., *Strongyloides* spp., and *Microsporidia* spp. [44].

Donor screening in some regions is limited, and commercial donors may come from low socio-economic backgrounds. Endemic infections, such as tuberculosis, malaria, Chagas disease, and leishmaniasis, may occur more frequently than in unpaid or deceased donors [39,45]. High infection rates may stem from intraoperative, hospital-acquired infections, or those originating from the donor, as well as infections acquired during the post-transplant period in endemic areas [31]. Urinary tract infections and pneumonia are frequently observed [37].

Countries where TT takes place are typically tropical or subtropical regions with a high prevalence of endemic infections, including malaria and tuberculosis. Additionally, these regions often have a high frequency of pathogens such as HIV, HBV, and HCV. The increased number of infections can also be attributed to inadequate screening of donors for infectious diseases and poor hygienic and operational conditions. Moreover, insufficient patient education regarding post-transplant infection risks and the lack of prophylactic measures against opportunistic infections may contribute to higher infection rates [38].

In cases of transplant tourism, patients often fail to follow standard travel guidelines for tropical and subtropical areas, including malaria prophylaxis. In 2019, guidelines were published regarding the management of organ transplant recipients due to potential medical issues encountered during travel [46].

In the patient described in this case, differential diagnosis did not initially consider infectious etiology, which was then deemed highly probable due to the patient’s foreign travels. Given the time spent in Pakistan, in addition to the pathogens previously mentioned, gastrointestinal infections caused by *Campylobacter jejuni*, *Escherichia coli*, *Shigella* spp., *Giardia intestinalis*, *Entamoeba histolytica*, *Cryptosporidium* spp., *Ascaris lumbricoides*, *Ancylostoma duodenalis*, *Strongyloides stercoralis*, *Trichuris trichiura*, *Enterobius vermicularis*, as well as rotaviruses, astroviruses, and noroviruses, should have been considered. In the described case, improper cyclosporine dosing was identified as the sole cause of impaired kidney functions in the transplanted organ.

In summary, commercial kidney transplantation from living donors, due to its illegal nature, is typically performed under substandard sanitary conditions, with only short-term postoperative monitoring and inadequate infection control measures. This situation significantly increases the risk of infectious complications, including bacterial, viral, parasitic, and fungal infections. Of particular concern are blood-borne viral infections such as CMV and HBV, as well as fungal infections caused by *Aspergillus fumigatus* and various species of Zygomycetes.

Based on data from the scientific literature, graft survival in patients experiencing infectious complications ranges from one month to three years, with an average of approximately 300 days. Additionally, mortality rates are substantially elevated, reaching around 47%. Therefore, clinicians should maintain a high level of vigilance for infectious complications, including atypical infections, in patients who have undergone transplantation abroad. Furthermore, TT should be mitigated by expanding the pool of deceased donors and improving programs related to living donors.

## Figures and Tables

**Table 1:** Laboratory studies and treatment of patient following commercial kidney transplantation in Pakistan (LOS, day of stay in the hospital; HGB, hemoglobin; Cr, creatinine; GFR, glomerular filtration rate; CRP, C-reactive protein; CsA, cyclosporin; TAC, tacrolimus; RV, reference values).

LOS	Laboratory Parameters				Treatment	
	HGB (mmol/L) RV: 8.5–11.0	Cr (mg/dL) RV: 0.7–1.2	GFR (mL/min/1.73 m^2^) RV: 80.0–120.0	CRP (mg/L) RV: <5	CsA (ng/mL)	TAC (ng/mL)
1	8.3	2.98	31	12	663	
2	7.4	2.65	24			
5	6.6	2.02	33		190	
8	5.6	3.72	16		Withdrawal	
9		4.98	11			13
10	5.5	4.54	12	14		
11		5.36	10			
12		5.83	9			16.8
15	5.4	4.50	12	8.43		
18	6.2	3.40	18			
21	6.7	2.56	25			11.7
24	7.1	2.37	27			

**Table 2:** Bacterial, viral, and fungal infections in patients following commercial kidney transplantation (M, male; F, female; CMV, cytomegalovirus; HBV, hepatitis B virus).

Age Sex	Residence	Location, Year	Symptoms Onset After Kidney Transplant Surgery	Pathogen	Graft Survival	Outcome	Reference
	Poland	Pakistan, 2019	2 weeks	Extended-spectrum β-lactamase-producing *Escherichia coli*	Yes	Alive	This study
61 M	USA	Pakistan	24 days	New Delhi metallo-beta-lactamase-1-producing *Enterobacter cloacae*, extended spectrum beta-lactamase-producing *Escherichia coli*, *Rhizopus arrhizus*	Necrotic kidney allograft	Alive	[22]
	USA	China or Pakistan or Philippines or India, 2001 or 2007		Resistant *Acinetobacter baumannii*	Two months	Died	[23]
	USA			HBV, *Mycobacterium tuberculosis*	26 months	Died	
46 M	USA	Pakistan		Cytomegalovirus, *Escherichia coli*, *Clostridioides difficile*	45 days	Alive	[13]
49 F	Australia	Lebanon, 2002		*Aspergillus* spp., multi-resistant *Pseudomonas aeruginosa*	1 month		[24]
66 M	USA	Pakistan, 2006		*Aspergillus flavus*	Yes	Alive	[25]
41 M	Saudi Arabia	Pakistan, 1999		Various species of Zygomycetes	Yes	Died (5 months)	[26]
48 W	Saudi Arabia	Philippines, 2001		*Aspergillus fumigatus*	-	Died (5 months)	[26]
35 W	USA	China		CMV	122 days	Alive	[13]
33 W	USA	China		HBV	154 days	Died	
75 M	Australia	China, 1993		CMV, HBV	12 months (death with a functioning graft)	Died	[24]
52 M		India		HBV		Died	
41 M		China, 2004		CMV	4 months	Alive	
43 M		China, 2002		Varicella zoster virus	2 years	Alive	
66 F		Eastern Europe, 2000		CMV	3 years	Alive	

## Data Availability

The data presented in this study are available on request from the corresponding author.
